# A Nodule-Localized Small Heat Shock Protein GmHSP17.1 Confers Nodule Development and Nitrogen Fixation in Soybean

**DOI:** 10.3389/fpls.2022.838718

**Published:** 2022-03-09

**Authors:** Zhanwu Yang, Hui Du, Jingyi Sun, Xinzhu Xing, Youbin Kong, Wenlong Li, Xihuan Li, Caiying Zhang

**Affiliations:** ^1^State Key Laboratory of North China Crop Improvement and Regulation, Hebei Agricultural University, Baoding, China; ^2^North China Key Laboratory for Crop Germplasm Resources of Education Ministry, College of Agronomy, Hebei Agricultural University, Baoding, China

**Keywords:** small heat shock proteins (sHSPs), soybean nodule, biological nitrogen fixation (BNF), peroxidase activity, molecular breeding

## Abstract

Small heat shock proteins (sHSPs) are ubiquitous proteins present in all organisms. The sHSPs are not only upregulated under heat shock as well as other stresses but also are expressed in unstressed cells, indicating quite diverse functions of sHSPs. However, there is little known about the role of sHSPs in nodulation and nitrogen fixation in soybean. In this study, we cloned a candidate protein of sHSP, GmHSP17.1, from proteome of nodule and analyzed its function in soybean nodulation. We found that GmHSP17.1 was a cytosolic protein and preferentially expressed during nodule development. An overexpression of *GmHSP17.1* in composite transgenic plants showed increases in nodule number, fresh weight, nodule size, area of infection cells, and nitrogenase activity, and subsequently promoted the content of nitrogen and growth of soybean plants. While *GmHSP17.1* RNA interference (RNAi) lines showed significantly impaired nodule development and nitrogen fixation efficiency. Through liquid chromatography-tandem mass spectrometry (LC-MS/MS), GmRIP1 was identified as the first potential target of GmHSP17.1, and was shown to be specifically expressed in soybean nodules. The interaction between GmHSP17.1 and GmRIP1 was further confirmed by yeast-two hybrid (Y2H), bimolecular fluorescence complementation (BiFC) *in vivo* and pull-down assay *in vitro*. Furthermore, peroxidase activity was markedly increased in *GmHSP17.1* overexpressed nodules and decreased in RNAi lines. As a result, the reactive oxygen species (ROS) content greatly decreased in *GmHSP17.1* overexpression lines and increased in suppression lines. Taken together, we conclude that GmHSP17.1 plays an important role in soybean nodulation through interacting with GmRIP1. Our results provide foundation for studying the mechanism of nitrogen fixation and for the genetics improvement of legume plants.

## Introduction

Legumes could obtain nitrogen source partially through biological nitrogen fixation (BNF). BNF is occurred in nodules of legumes, a specialized root structures harboring the bacteria. In root nodules, the bacteria finally differentiate into bacteroids, which are surrounded by a plant root derived membrane, peribacteroid membrane, to form the so-called symbiosome, and then atmospheric N_2_ is fixed by the nitrogenase enzyme complex in the bacteroids into the forms of ammonia (Udvardi and Day, 1997; Ferguson et al., 2010). Therefore, BNF is thought to be an alternative way of chemical nitrogen fertilizer in the agroecosystems. BNF is a highly energy-consuming process and regulated by complex molecular dialog. Legume plants have evolved strategies to negatively control nodule numbers, called autoregulation of nodulation (AON) pathway to balance the nitrogen gains and energy consumption (Suzuki et al., 2008; Reid et al., 2011; Suzaki and Nishida, 2019; Isidra-Arellano et al., 2020). In addition, legume nodulation is regulated by nitrogen source, when there is sufficient available nitrogen in the soil, legume plants will cease the symbiosis with *Rhizobium* to save the energy (Nishida and Suzaki, 2018; Ferguson et al., 2019). Therefore, it is a more economic and effective strategy to increase the ability of nitrogen fixation for reducing the application of nitrogen fertilizer in legumes.

In recent decades, considerable progress has been made to explore novel genes regulating the symbiotic signaling pathway in legume plants (Marx et al., 2016; Qiao et al., 2016; Yuan et al., 2016). An important finding these days in soybean was reported that light-induced factors GmSTF3/4 and GmFTs moved from shoots to roots to regulate nodule organogenesis, which integrated the aboveground light signals with underground symbiotic signaling and provided approaches to enhance the balance of carbon and nitrogen in the biosphere (Wang et al., 2021). In legume nodules, leghemoglobins (LgHbs) enable the endosymbiotic nitrogen fixation by binding to O_2_ to protect O_2_-sensitive nitrogenase. In nodules of *Medicago truncatula*, NIN-like protein (NLP) transcription factors NLP2, and NIN highly expressed and directly interacted with leghemoglobin genes to finally regulate the nitrogen fixation (Jiang et al., 2021). All these research provide insights into the molecular mechanism by which the legume plants regulate the nodule development and nitrogen fixation.

Small heat shock proteins (sHSPs) are virtually ubiquitous and diverse proteins present in plants. sHSPs can be divided into 11 conserved subfamilies, such as six (CI–CVI) cytosolic subfamilies and five subfamilies that localize to organelles, such as mitochondria, chloroplast, endoplasmic reticulum (ER), and peroxisome (Sun et al., 2002; Waters and Rioflorido, 2007; Bondino et al., 2012; Waters, 2013; Carra et al., 2017; Waters and Vierling, 2020). Extensive studies have shown that sHSPs not only highly expressed during heat shock stresses, but also in other environmental stresses, such as oxidative stress, drought, cold, and heavy metals (Sun and MacRae, 2005; Sun et al., 2016, 2020, 2021). Furthermore, sHSPs was also found to be involved in chloroplast development, seed germination, and fruit maturation (Zhong et al., 2013; Zhang et al., 2018; Ma et al., 2019). A chloroplast heat shock protein, AsHSP26.8, in creeping bentgrass (*Agrostis stolonifera L.*) was shown to have a role in modulating the plant growth and abiotic stress response, such as heat, salt, and drought stress (Sun et al., 2021). In addition, another chloroplast heat shock protein in *Arabidopsis thaliana*, Hsp17.8, functioned in the targeting of chloroplast outer membrane proteins (Kim et al., 2011). GhHSP24.7, a mitochondrial matrix-localized sHSP, regulated cotton seed germination in a temperature-dependent manner (Ma et al., 2019).

Recently, our research team for the first time reported that sHSP, GmHSP17.9, identified in the proteome of nodules confers nodule development and symbiotic nitrogen fixation *via* interacting with sucrose synthase GmNOD100 in soybean (Yang et al., 2021). In the meantime, another sHSP, named GmHSP17.1 (*Glyma.06g157800*), was found to be preferentially expressed in nodules. In this study, we generated composite transgenic soybean plants either overexpression or suppression of *GmHSP17.1* to explore the function of *GmHSP17.1* in nodules. Furthermore, we found that GmHSP17.1 directly interacted with a peroxidase, GmRIP1, to regulate the nodule development. Our findings revealed a molecular mechanism of sHSPs involving in the nodule development of soybean and expand our knowledge on the understanding of sHSPs.

## Materials and Methods

### Plant Materials and Growth Conditions

Soybean ecotype Williams 82 was used for the phenotypic and functional analysis in this study. Healthy soybean seeds were chlorinated and planted into vermiculite after 3-day germination. Furthermore, 7-day seedlings were inoculated with *Bradyrhizobium diazoefficiens* USDA110 and grown in a growth chamber under a 16 h light:8 h dark cycle at 28°C. The seedlings were watered with nitrogen-free nutrient solution (containing 2.5 mM K_2_SO_4_, 2 mM MgSO_4_•7H_2_O, 1 mM KH_2_PO_4_, 0.15 mM FeCl_2_, 1.5 mM CaSO_4_2•H_2_O, 46 μM H_3_BO_3_, 9.1 μM MnCl_2_•4H_2_O, 0.75 μM ZnSO_4_, 0.5 μM CuSO_4_, 0.11 μM MoO_3_, 9.4 × 10^–2^ μM CoCl_2_•6H_2_O).

The soybean plants were harvested at 28 days post inoculation (dpi) for measuring the fresh weight, dry weight, plant height, and N content of shoot. Nodules were separately harvested for measuring nitrogenase activity, nodule number, and fresh weight. For quantitative real-time PCR (qRT-PCR) analysis, nodules were harvested at 10, 17, 21, 28, and 35 dpi. All tissues were frozen in liquid nitrogen and stored at –80°C for further mRNA and protein analyses.

### Gene Expression Analysis by Quantitative Real-Time PCR

Total RNA was extracted using the RNAprep Pure Plant Kit (Tiangen, Beijing, China), and cDNA was synthesized using a PrimeScript™ RT reagent Kit with gDNA Eraser (Takara, Otsu, Shiga Prefecture, Japan). SYBR Premix EX Taq™ (Takara, Otsu, Shiga Prefecture, Japan) was used for qRT-PCR analysis by a CFX96™ real-time system (Bio-Rad, Berkeley, CA, United States). The qRT-PCR reaction conditions were: 95°C 30 s, 95°C 10 s, 56°C 15 s, and 72°C 10 s. The fold change in the expression of each sample was standardized using *GmActin11* gene and analyzed by the 2^–ΔΔCT^ method (Livak and Schmittgen, 2001). Lowercase letters represent statistically significant differences (*p* < 0.05) according to Tukey’s HSD test; asterisks indicate statistically significant differences according to Student’s *t-*test (two-tailed) (**p < 0.01). All experiments were repeated at least three times. The primers of qRT-PCR are listed in Supplementary Table 1.

### Purification and Chaperone Activity of GmHSP17.1 *in vitro*

A *GmHSP17.1* protein was cloned into pET-28a (+) vector and introduced into the *Escherichia coli* strain, BL21 (DE3) (EMD Chemicals Inc., Gibbstown, NJ, United States). GmHSP17.1 proteins were induced by 0.5 mM isopropyl-β-d-thiogalactoside for 4 h at 28°C and purified by Ni-Agarose Resin (Lot 01376/10531, CWBIO, China) according to the manufacturer’s instructions. Green fluorescent proteins (GFPs) were purified in the same way as GmHSP17.1 and used as negative controls in this experiment. The chaperone activity of GmHSP17.1 was performed by measuring the chemically induced aggregation of insulin (from bovine pancreas, Sigma-Aldrich, St. Louis, MO, United States) and thermal aggregation of malate dehydrogenase (MDH; from porcine heart, Sigma-Aldrich) according to previous protocols (Yang et al., 2021).

### Histochemical β-Glucuronidase Staining Analysis

Promoter of *GmHSP17.1*, 2.4-kb length upstream of ATG, was cloned into pBI121 vector to generate pHSP17.1::β-glucuronidase (GUS) construct. pHSP17.1::GUS was transformed into *Agrobacterium rhizogenes* strain K599 for further hairy roots transformation, as described previously (Kim et al., 2013). The nodules on the transgenic hairy root were harvested at 10, 17, 21, and 28 dpi for GUS staining. GUS staining was performed as described previously (Luo et al., 2013; Zhong et al., 2013).

### Construction of *GmHSP17.1* Overexpression and RNA Interference Cassettes and Soybean Hairy Root Transformation

The full-length open reading frame (ORF) of *GmHSP17.1* was cloned into pCamE-GFP vector between *Sal*I and *Bam*HI enzyme sites for an overexpression analysis. For the RNAi constructs, about 200 bp fragment specific to *GmHSP17.1* was inserted between *Bam*HI and *Kpn*I, *Spe*I and *Sac*I, respectively, in pTCK-303-GUS vector, as described previously (Du et al., 2016; Wang et al., 2020). Empty vectors alone were used as negative controls. Then, the vectors were transformed into hairy roots through *Agrobacterium rhizogenes* strain K599, as described previously (Kim et al., 2013). GFP fluorescence signal and GUS staining were used to identify the positive transgenic hairy roots.

### Observation of Infection Cells by Toluidine Blue Staining

For the observation of infection cells, three nodules randomly selected at 28 dpi were fixed in formaldehyde-acetic acid solution for 24 h at 4°C with three independent experiments. After embedding in paraffin, 5 μm sections were prepared using a microtome (RM2016). After dewaxing, the nodule sections were stained with 0.1% Toluidine Blue and images of three sections for each nodule were captured with a scanner (Pannoramic DESK, P-MIDI, P250). The percentage of area of infection cells to total cells in a nodule section and surface area of 100 infection cells were calculated with Image-Pro Plus 6.0 software.

### Identification of Interacting Proteins of GmHSP17.9 in Nodules

The coding DNA sequence (CDS) of *GmHSP17.1* was cloned into the pET-28a-Avi (+) vector to generate His-GmHSP17.1-Avi fusion protein, which contains an additional Avi-tag at the C-terminal end (Du et al., 2015). Then, the constructed vector was transformed into the *E. coli* strain, BL21 which was pre-transformed with BirA for biotinylation (Tirat et al., 2006). Fusion protein of GmHSP17.1 was induced by 0.5 mM IPTG for 4 h at 28°C and purified by affinity chromatography using streptavidin agarose resin (Thermo Fisher Scientific, Waltham, MA, United States). GFP was used as a negative control. The total protein of soybean nodule was extracted with Plant Protein Extraction Reagent (CWBIO, Beijing, China) and incubated with purified GmHSP17.1 protein at 4°C. The targeted proteins were separated by affinity chromatography, and then, purified three times with buffer solution. The purified proteins were isolated by electrophoresis with 12% sodium dodecyl sulfate-polyacrylamide gel electrophoresis (SDS-PAGE) gel and were further analyzed by liquid chromatography-tandem mass spectrometry (LC-MS/MS) (Supplementary Table 2).

### Subcellular Localization and Bimolecular Fluorescence Complementation Analysis

The CDSs of *GmHSP17.1* or *GmRIP1* was cloned into 326-GFP vector to generate *GmHSP17.1-*GFP of *GmRIP1-*GFP, respectively, for subcellular localization. GFP fluorescence was captured using a confocal microscope (Fvi10, OLYMPUS, Tokyo, Japan). The CDSs of *GmHSP17.1* and *GmRIP1* were cloned into the p326YFP^N^ and p326YFP^C^, respectively, to generate GmHSP17.1-YFP^N^, GmHSP17.1-YFP^C^, GmRIP1-YFP^C^, and GmRIP1-YFP^N^ for BiFC assay. The constructed vectors were transformed into *Arabidopsis* protoplasts, according to the methods described previously (Yoo et al., 2007). Yellow fluorescent protein (YFP) fluorescence was observed using a confocal microscope (Fvi10, OLYMPUS, Tokyo, Japan).

### Yeast Two-Hybrid Assay

The interaction between GmHSP17.1 and GmRIP1 was verified by a yeast two-hybrid experiment using the Matchmaker Gold Yeast Two-Hybrid System according to the instructions (Clontech, 630489, Mountain View, CA, United States). *GmHSP17.1* was cloned into pGBKT7 as bait (pGBKT7-*GmHSP17.1*), while the CDS of *GmRIP1* was cloned into pGADT7 as prey (pGADT7-*GmRIP1*). The pGBKT7-*GmHSP17.1* and pGADT7-*GmRIP1* were co-transformed into yeast-two hybrid (Y2H) Gold cell, and then incubated on SD/-Leu-Trp plates at 30°C for 3 days. The positive clones were then transferred to SD/-His-Ade-Leu-Trp plates and SD/-His-Ade-Leu-Trp + X-α-Gal + AbA (Takara, Otsu, Shiga Prefecture, Japan) and incubated at 30°C for 5 days to confirm the interaction. In addition, pGBKT7-53 and pGADT7-T were used as positive controls, and pGBKT7-Lam and pGADT7-T as negative controls. The primers used in the Y2H assay are listed in Supplementary Table 1.

### Pull-Down Assay

The CDS of *GmRIPP1* was cloned into pET-28a (+) vector to generate His-tag fusion protein (His-GmRIP1), and the CDS of *GmHSP17.1* was cloned into the pET-28a-Avi (+) vector to generate His-GmHSP17.1-Avi fusion protein. These constructs were transformed into the *E. coli* strain BL21 for protein induction. His-GmHSP17.1-Avi was purified using a streptavidin agarose resin, and then incubated with the total proteins isolated from BL21 expressing His-GmRIP1. Finally, the western blot was performed with anti-His monoclonal antibody (Invitrogen, Carlsbad, CA, United States) (Du et al., 2010). The primers used in pull-down assay are listed in Supplementary Table 1.

### Measurement of Nitrogenase Activity and N Content

Nitrogenase activity was measured by acetylene reduction assay according to the protocol described previously (Oh et al., 2001). The dried nodule samples were nitrate-boiled and the N content was measured using Semimicro-Kjeldahl determination method in a nitrogen analyzer as described previously (Wang et al., 2020).

### Measurement of Peroxidase Activity and Reactive Oxygen Species Content in Nodules

Peroxidase activity of nodules was measured using a Peroxidase Activity Detection Kit (Solarbio, China) according to the manufacturer’s instructions. The content of reactive oxygen species (ROS) of nodules was measured using a Plant ROS Kit (Chenglin, Beijing, China), according to the manufacturer’s instructions.

### Statistical Methods

Statistical analyses were performed using GraphPad Prism 7 software.

## Results

### *GmHSP17.1* Was Preferentially Expressed in Nodules of Soybean

**FIGURE 1:**
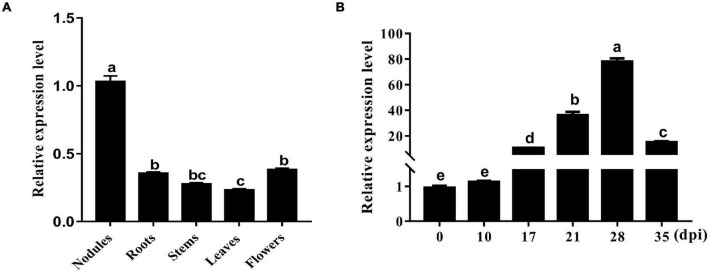
Expression pattern of *GmHSP17.1* in different organs of soybean. **(A)** Transcript accumulation of *GmHSP17.1* in soybean nodules at 28 dpi, roots, stems, leaves, and flowers. **(B)** The expression profiles of *GmHSP17.1* in rhizobia-inoculated roots (0 dpi) and nodules (10, 17, 28, and 35 dpi). The relative expression value was normalized based on the expression of *GmActin11* (*Glyma.18g290800*) used as reference gene. Lowercase letters represent statistically significant differences (*p* < 0.05) according to Tukey’s HSD test. All experiments were repeated at least three times. Dpi, days post inoculation.

**FIGURE 2:**
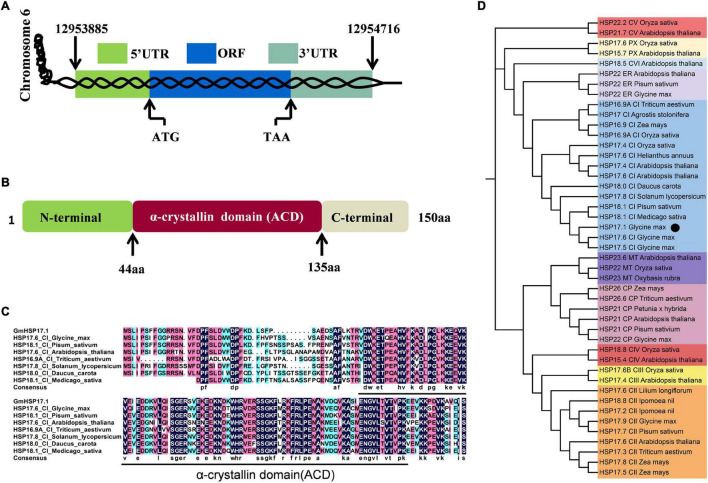
Bioinformatics analysis of GmHSP17.1. **(A)** A genome sequence analysis of *GmHSP17.1*. **(B)** The domain structure of GmHSP17.1 protein. α-crystallin domain (ACD) (44–135 aa), N-(1–43 aa), and C-(136–150 aa) terminal sequences are indicated. **(C)** The alignment of the amino acid sequences of GmHSP17.1 and other CI subfamily members in *Glycine max*, *Pisum sativum*, *Arabidopsis thaliana, Triticum aestivum, Solanum lycopersicum, Daucus carota*, and *Medicago sativa*. The alignment was performed using DNAMAN. The black line indicated the ACD domain. **(D)** Phylogenetic tree analysis of GmHSP17.1. Phylogenetic tree was conducted by the MEGA7 software. Accession numbers of the sHSPs were: *Glycine max* (HSP17.9, NP_001346002; HSP17.5, NP_001362775.1; HSP17.6, NP_001347279.1; HSP22, NP_001347237.1; HSP22, NP_001236586.2), *Agrostis stolonifera* (HSP17, ALR99802.1), *Arabidopsis thaliana* (HSP17.6, NP_175759.1; HSP17.4, NP_190209.1; HSP17.6, NP_196763.1; HSP17.4, NP_001323264.1; HSP15.4, NP_193918.1; HSP21.7, NP_568810.1; HSP18.5, NP_179521.1; HSP15.7, NP_198583.1; HSP21, NP_194497.1; HSP23.6, NP_194250.1; HSP22, NP_192763.1), *Daucus carota* (HSP18.0, P27397.1), *Helianthus annuus* (HSP17.6, XP_021973842.1), *Solanum lycopersicum* (HSP17.8, NP_001266045.1), *Medicago sativa* (HSP18.1, P27879.1), *Oryza sativa* (HSP16.9A, XP_015625199.1; HSP17.4, XP_015631117.1; HSP18.8, XP_015645510.1; HSP22.2, XP_015638251.1; HSP17.6, XP_015641984.1; HSP22, XP_015626255.1; HSP17.6B, XP_015623982.2), *Zea mays* (HSP16.9, ACG40361.1; HSP17.5, P24631.1; HSP17.8, NP_001105954.1; HSP26, NP_001105583.1), *Triticum aestivum* (HSP26.6, Q00445.1; HSP17.3, CAA41218.1; HSP16.9A, XP_044445338.1), *Pisum sativum* (HSP18.1, P19243.1; HSP17.7, P19242.1; HSP21, P09886.1; HSP22, P19244.1), *Ipomoea nil* (HSP18.8, Q01545.1; HSP17.2, Q01544.1), *Lilium longiflorum* (HSP17.6, BAA04840.1), *Petunia x hybrid* (HSP21, P30222.1), and *Oxybasis rubra* (HSP23, P11890.1). The black dot indicates the position of GmHSP17.1.

An sHSP, GmHSP17.1, was identified in the proteome of mature nodules of soybean, and the expression profile in various organs showed that the transcript abundance of *GmHSP17.1* in nodules was about 2.7 times higher than in flowers, followed by roots, stems, and leaves (Figure 1A). Next, the transcript accumulation of *GmHSP17.1* was determined *via* qRT-PCR in soybean roots inoculated with *Bradyrhizobium diazoefficiens* USDA110, we found that *GmHSP17.1* was induced more strongly in nodules. The transcript of *GmHSP17.1* increased gradually during the nodule development until 28 dpi and decreased at 35 dpi (Figure 1B). *GmHSP17.1* gene (*Glyma.06g157800*) was located on chromosome 6, and a gene model analysis revealed that *GmHSP17.1* had only one exon and no intron, with a length of 832 bp for the predicted mature transcript, 106 bp for the 5′ untranslated region (UTR), 273 bp for the 3′ UTR, and an ORF of 453 bp (Figure 2A). The ORF of *GmHSP17.1* encoded a predicted protein of 150 amino acid residues with a conserved α-crystallin domain, a defined domain for sHSPs family in plant species (Figures 2B,C). Furthermore, phylogenetic tree showed that GmHSP17.1 belonged to the CI subfamily of sHSPs (Figure 2D), which were reported to be localized in cytoplasm (Waters, 2013). sHSPs are known to be molecular chaperones in living cells (Haslbeck and Vierling, 2015; Carra et al., 2017). The molecular chaperone activity of *GmHSP17.1* was confirmed by insulin and MDH as conventional substrates. The results indicated that *GmHSP17.1* efficiently prevented chemically induced aggregation of insulin by DTT and thermal aggregation of MDH at 45°C while no holdase activity was detected in the presence of GFP (Supplementary Figures 1A,B).

**FIGURE 3:**
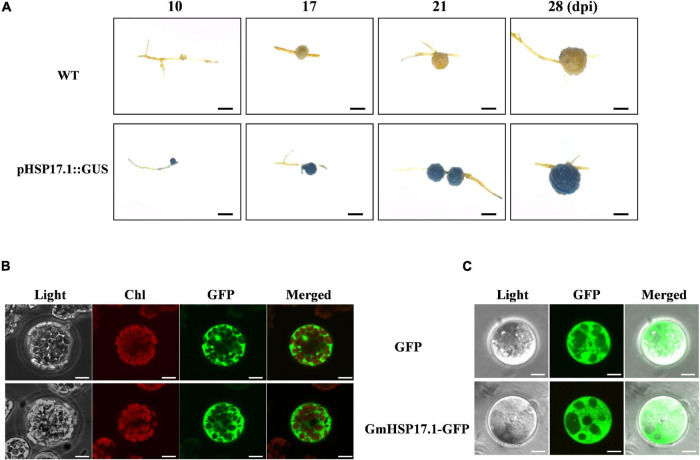
Promoter and subcellular localization analysis of *GmHSP17.1*. **(A)** β-glucuronidase (GUS) staining of transgenic composite soybean roots and nodules expressing pHSP17.1::GUS at different developmental stages. Three independent experiments were performed, and images from one representative experiment were shown here (*n* > 10). Scale bar = 1 mm. **(B)** Subcellular localization of GmHSP17.1 in *Arabidopsis* protoplasts. *GmHSP17.1-GFP* construct was transformed into *Arabidopsis* protoplasts and green fluorescent protein (GFP) fluorescence was observed in the cytoplasm of protoplasts. GmHSP17.1-GFP: GmHSP17.1 was fused with GFP. Scale bars = 10 μm. **(C)** Subcellular localization of GmHSP17.1 in soybean root protoplasts. The protoplasts were isolated from transgenic hair roots overexpressing GmHSP17.1-GFP. GFP fluorescence was observed by a confocal fluorescence microscope. The free GFP (empty vector) was used as control. Scale bars = 5 μm.

To further analyze the expression pattern of *GmHSP17.1* in soybean nodules, 2,400 bp promoter sequence upstream of start codon was fused with the reporter gene *GUS*, and the resulting construct *GmHSP17.1pro::GUS* was then used to generate transgenic composite hairy roots and nodules. Histochemical staining analysis indicated that *GmHSP17.1* was preferentially expressed during nodule development, consistent with the qRT-PCR results (Figure 3A). To further understand the subcellular localization of GmHSP17.1, *GmHSP17.1* was fused with reporter gene *GFP* (*GmHSP17.1-GFP*) driven by the CaMV 35S promoter. *GmHSP17.1-GFP* was then transfected into *Arabidopsis* protoplasts, and strong GFP expression signal was captured in the cytoplasm, in agreement with GFP control (Figure 3B). In the meantime, the subcellular localization of GmHSP17.1 was also verified in the protoplasts of soybean transgenic hairy roots. A strong GFP fluorescence signal was also observed in the cytoplasm of root protoplasts, as well as cytosolic protein GFP used as a marker (Figure 3C). Taken together, these data showed that *GmHSP17.1* was preferentially expressed in nodules, indicating an important role in nodule development in soybean.

### Altered Expression of *GmHSP17.1* Affected Nodulation, Biological Nitrogen Fixation Capacity, and Plant Growth in Soybean

**FIGURE 4:**
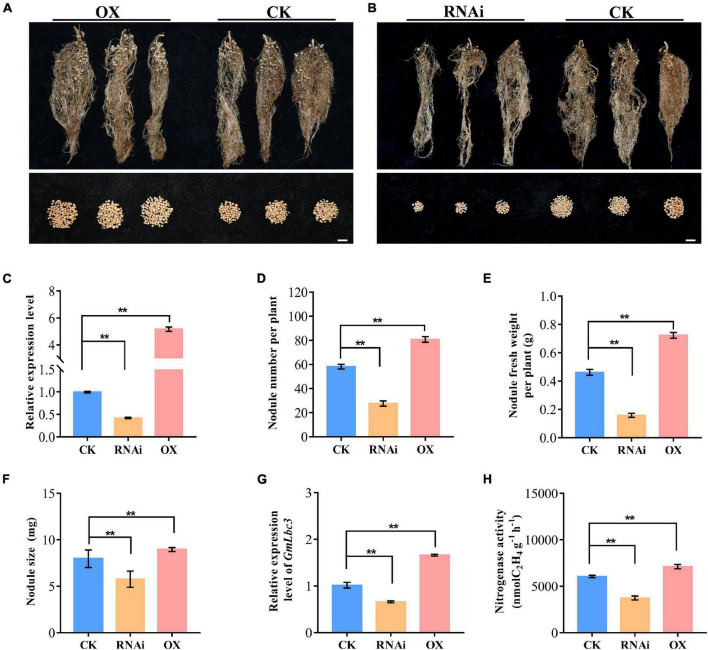
Phenotypic analysis of nodulation of transgenic composite lines overexpressing (OX) and RNA interference (RNAi)-silenced *GmHSP17.1*. **(A,B)** Growth performance of nodules at 28 dpi. Scale bar = 1 cm. **(C)** Relative expression level of *GmHSP17.9* in nodules at 28 dpi. **(D)** Nodule number. **(E)** Nodule fresh weight. **(H)** Nitrogenase activity measured by the acetylene reduction assay. **(F)** Nodule size. **(G)** The relative expression of *GmLbc3.* CK refers to transgenic plants carrying empty vector. Asterisks indicate statistically significant differences according to Student’s *t*-test (two-tailed) (**p < 0.01), all experiments were repeated at least three times, *n* = 10.

**FIGURE 5:**
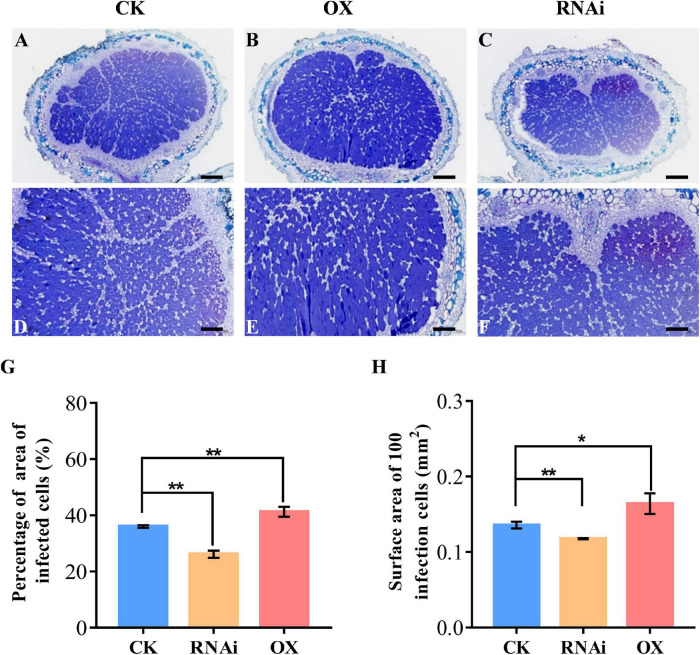
Cross sections of toluidine blue-stained nodule of *GmHSP17.1* OX and RNAi lines. **(A,D)** Toluidine blue staining of nodules expressing CK empty vector. **(B,E)** Toluidine blue staining of nodules overexpressing of *GmHSP17.1*. **(C,F)** Toluidine blue staining of nodules of *GmHSP17.1* RNAi lines. **(G)** Percentage of area of infection cells to all cells in one nodule section. **(H)** Surface area of 100 infection cells. **(A–C)** Scale bar = 200 μm, **(D–F)** scale bar = 100 μm. Asterisks indicate statistically significant differences according to Student’s *t*-test (two-tailed) (*p < 0.05; **p < 0.01), these experiments were repeated at least three times and similar results were obtained; *n* = 3.

To investigate the function of *GmHSP17.1* in nodule development and BNF capacity in soybean, we performed phenotypic analyses of transgenic composite soybean plants either overexpressing or suppressing of *GmHSP17.1* (Figures 4A,B). The success of transformation in transgenic hairy roots was determined by qRT-PCR, and we found that the expression of *GmHSP17.1* in overexpression lines was 4-fold of the expression in control lines, while in suppression lines, the expression of *GmHSP17.1* was reduced by 57.7% (Figure 4C). The nodule number, fresh weight, and nodule size increased by 38.8, 56.6, and 12.5% in overexpression lines, while decreased by 52.5, 65.6 and 27.6% in suppression lines, respectively, in comparison with control lines (Figures 4D,F). Consistently, the expression of leghemoglobin gene *GmLbc3* was also increased in overexpression lines and decreased in the RNAi lines (Figure 4G). As a result, nitrogen fixation efficiency was markedly affected due to increased and decreased nitrogenase activity in *GmHSP17.1* overexpressed and RNAi nodules, respectively (Figure 4H). Furthermore, the infection cells of nodules were examined by toluidine blue staining and we found the *GmHSP17.1* overexpressed nodules displayed larger infection cells and infection areas, while RNAi nodules showed smaller infection cells and infection zone, compared with that of control lines (Figure 5).

In addition, soybean plant growth was significantly influenced due to an altered expression of *GmHSP17.1* in transgenic composite plants (Supplementary Figure 2A). An overexpression of *GmHSP17.1* resulted in the increase of 48.4, 33.2, 33.8, and 8.2% in plant height, shoot fresh- and dry weight, and N content; on the other hand, the suppression of *GmHSP17.1* showed inhibition of plant height, shoot fresh- and dry weight, and N content by 34.3, 24.4, 26.0, and 27.3%, respectively, compared with that of control lines (Supplementary Figures 2B–E). Taken together, these results indicate that *GmHSP17.1* affect the nodule development and nitrogen fixation, and subsequently along with the plant growth.

### *GmHSP17.1* Directly Interacts With GmRIP1 Peroxidase

**FIGURE 6:**
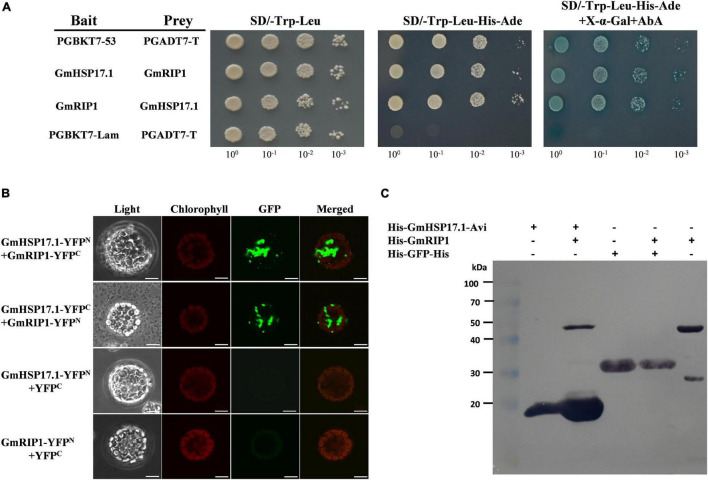
GmHSP17.1 interacts with GmRIP1. **(A)** Interaction between GmHSP17.1 and GmRIP1 in yeast. Positive yeast strains were selected on SD/-Trp-Leu medium and further verified on SD/-Tru-Leu-His-Ade medium containing 125 ng/ml AbA and 40 μg/ml X-α-Gal. **(B)** bimolecular fluorescence complementation (BiFC) analysis of interaction between GmHSP17.1 and GmRIP1 in *Arabidopsis* protoplasts. Fluorescence signal could only be found in the cytoplasm of *Arabidopsis* protoplasts transformed with GmHSP17.1-YFP^N^ and GmRIP1-YFP^C^ or GmHSP17.1-YFP^C^ and GmRIP1-YFP^N^, while there was no fluorescence signal in protoplasts with GmHSP17.1-YFP^N^ or GmRIP1-YFP^N^ co-expressed with empty vector YFP^C^. Scale bars = 10 μm. **(C)** Interaction between GmHSP17.1 and GmRIP1 *in vitro* by pull-down assay. Pull-down assay was performed using recombinant His-GmHSP17.1-Avi protein purified by streptavidin agarose resin and the total cell lysates of His-GmRIP1 and the western blot with anti-His antibody.

To further illuminate the molecular mechanism of GmHSP17.1 in nodules, interacting proteins of *GmHSP17.1* were isolated by LC-MS/MS (Supplementary Figure 3A). A peroxidase protein, homology of a rhizobium-induced peroxidase (Rip1) identified in *M. truncatula* (Goormachtig et al., 1995; Ramu et al., 2002), designated GmRIP1 was chosen as the first candidate of *GmHSP17.1* (Supplementary Table 2). The phylogenetic analysis showed that GmRIP1 belonged to class III peroxidases which include all secretory plant-specific peroxidases (Supplementary Figure 3B). In the meantime, we found that the peroxidase activity of nodules was increased during nodule development (Supplementary Figure 3C). These data indicated that peroxidases may have important roles in nodules. Next, the interaction between GmHSP17.1 and GmRIP1 was further confirmed by an independent Y2H assay. BD-GmHSP17.1 and AD-GmRIP1 or BD-GmRIP1 and AD-GmHSP17.1 constructs were co-transformed into Y2H cells, respectively, and positive colonies were selected on SD/-Trp-Leu-His-Ade + X-α-gal + AbA medium (Figure 6A). To verify the interaction between GmHSP17.1 and GmRIP1 *in vivo*, we performed the bimolecular fluorescence complementation (BiFC) analysis in *Arabidopsis* protoplasts. A strong YFP fluorescence signal was detected in the cytoplasm of *Arabidopsis* protoplasts expressing GmHSP17.1-YFP^N^ and GmRIP1-YFP^C^ or GmHSP17.1-YFP^C^ and GmRIP1-YFP^N^, whereas no YFP fluorescence was observed in the negative control combinations GmHSP17.1-YFP^N^/YFP^C^ and GmRIP1-YFP^N^/YFP^C^ (Figure 6B). In addition, the interaction between GmHSP17.1 and GmRIP1 was also confirmed by pull-down assay using recombinant purified proteins in *E. coli* (Figure 6C). Taken together, we conclude that GmHSP17.1 directly interacts with GmRIP1 *in vivo* and *in vitro*.

### GmRIP1 Was a Cytosolic Protein and Induced in Nodules of Soybean

**FIGURE 7:**
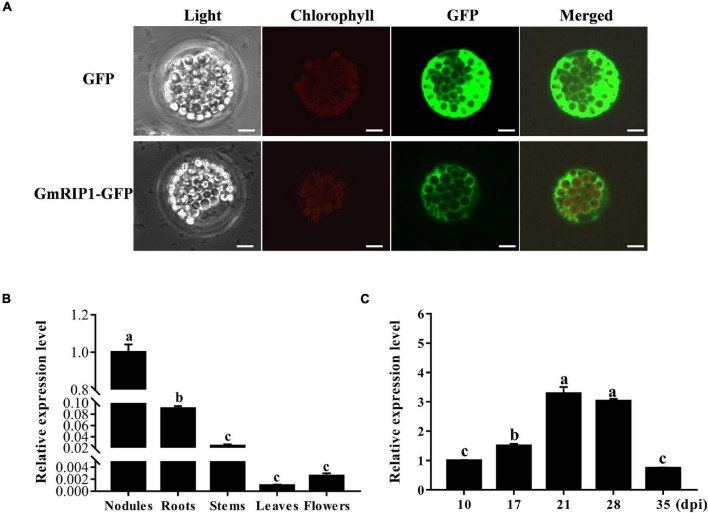
Subcellular localization and expression analysis of GmRIP1. **(A)** Subcellular localization of GmRIP1 in protoplasts of *Arabidopsis thaliana.* The fluorescence of GmRIP1-GFP was observed in the cytoplasm of *Arabidopsis* protoplasts. The free GFP (empty vector) used as control was distributed in both nucleus and cytoplasm. GmRIP1-GFP: GmRIP1 was fused with GFP. Scale bars = 10 μm. **(B)** Transcript accumulation of GmRIP1 in various organs of soybean. **(C)** Relative expression of GmRIP1 in nodules at different developmental stages. The relative expression value was normalized based on the expression of *GmActin11* (*Glyma.18g290800*) used as reference gene. Lowercase letters represent statistically significant differences (*p* < 0.05) according to Tukey’s HSD test; all experiments were repeated at least three times.

Peroxidases are important enzymes, acting as antioxidants, in plants that involves in the production and scavenging of ROS, such as superoxide radicals and H_2_O_2_ (Wang et al., 2015). In the nodulation process, ROS was produced in response to rhizobium infection and could be detected later in infection thread and infection zones of nodules (D’Haeze et al., 2003). Subcellular localization of GmRIP1 was determined in *Arabidopsis* protoplasts. GmRIP1-GFP fusion protein driven by the CaMV 35S promoter was transiently expressed in *Arabidopsis* protoplasts, and the expression of GmRIP1-GFP was detected in the cytoplasm (Figure 7A). Next, expression profiles of *GmRIP1* in various tissues of soybean were analyzed by qRT-PCR, and the data showed that *GmRIP1* was highly expressed in nodules and roots than in stem, leaf, and flower (Figure 7B). Expression of *GmRIP1* in different developmental stages of nodules was determined, and we found that *GmRIP1* was gradually increased during nodule growth (Figure 7C). All these data indicated that GmRIP1 played a key role in the nodulation in soybean.

### GmHSP17.1 Regulates Nodule Development Through Interacting With Peroxidase GmRIP1

**FIGURE 8:**
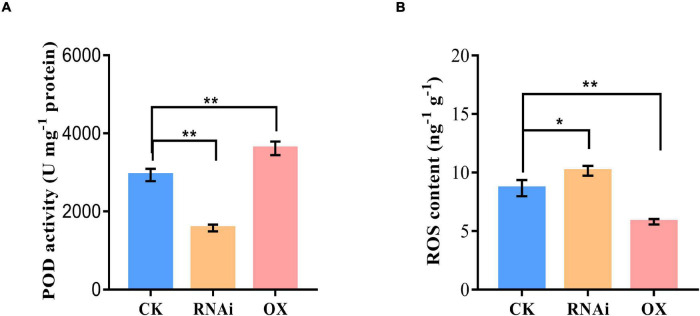
Peroxidase (POD) activity and reactive oxygen species (ROS) content measurement in nodule of *GmHSP17.1* OX and RNAi lines. **(A)** POD activity determination. **(B)** ROS content determination. Asterisks indicate statistically significant differences according to Student’s *t*-test (two-tailed) (*p < 0.05; **p < 0.01), all experiments were repeated at least three times.

To further confirm the interaction effect between GmHSP17.1 and GmRIP1, peroxidase activity of GmRIP1 was measured in *GmHSP17.1* overexpression and suppression lines, and we found that peroxidase activity increased by 63.3% in overexpression lines and decreased by 35.1% in suppression lines (Figure 8A). As a result, the content of ROS was significantly affected by the altered peroxidase activity. The content of ROS was greatly decreased by 7.8% in *GmHSP17.1* overexpression lines and increased by 25.9% in suppression lines (Figure 8B). Taken together, we conclude that GmHSP17.1 conferred nodule development and nitrogen fixation partially through the regulating peroxidase activity of GmRIP1.

## Discussion

Biological nitrogen fixation is a unique process in legume plants. In the past decades, numerous studies have focused on mining genes and its molecular mechanisms, and expected to endow the ability of nitrogen fixation in non-leguminous plants, and very few powerful functional genes associated with BNF were discovered. Therefore, it is the most objective to explore genes with clear molecular mechanisms for nodule development and BNF in the present. sHSPs are usually chaperone proteins not only involved in diverse stresses, but also in the plant development, such as in pollen, chloroplast, and seed development (Sun et al., 2002; Waters et al., 2008; Waters and Vierling, 2020). However, the role of sHSPs in nodule formation, development, and nitrogen fixation are largely known in legume plants especially in soybean, with only a few papers reported previously. PvNod22, a non-canonical HSP in the endoplasmic reticulum (ER), from a common bean (*Phaseolus vulgaris* L.), was involved in the infection thread progression during rhizobial infection, which was important for nodule organogenesis (Rodriguez-Lopez et al., 2019). In the nodules of cowpea (*Vigna unguiculata*), VuHSP17.7, an sHSP family class I protein, was highly induced by high-temperature stress in nodules, suggesting a role in signaling pathways under heat stress (Simoes-Araujo et al., 2008). In this study, the gene GmHSP17.1, encoding an sHSP in cytoplasm, was discovered and its expression revealed by qRT-PCR and promoter-GUS analysis in nodules indicated that *GmHSP17.1* was specifically expressed in nodules (Figures 1, 3). To further understand the function of *GmHSP17.1* in nodules, composite transgenic plants were generated and a series of experiments were conducted. The results indicated that the expression of *GmHSP17.1* was significantly associated with the number of nodules, nodule size, and also the activity of nitrogenase (Figures 4, 5). All the data suggested that *GmHSP17.1* was involved in nodule development and nitrogen fixation, and this finding was quite different from the function of other sHSPs studied in soybean.

To further elucidate the molecular mechanism of sHSPs underling its function, usually its target proteins were identified. HSP21 in *Arabidopsis*, cooperated with its *in vivo* target pTAC5 under heat stress to regulate proper chloroplast development (Zhong et al., 2013). Hsp17.8, in the chloroplast of *Arabidopsis*, acted as a cofactor of AKR2A in targeting membrane proteins to outer membranes of plastid under normal physiological conditions (Kim et al., 2011). Recently, IPN2, interacting protein of Nodulation Signaling Pathway 2 (NSP2), regulated root nodule symbiosis by binding to the IPN2-responsive *cis* element (IPN2-RE) of NIN promoter and activated NIN expression allowing nodulation in *Lotus japonicus* (Xiao et al., 2020). In the present study, we isolated GmRIP1, a peroxidase, was a potential target of GmHSP17.1 in soybean nodules by LC-MS/MS. Directly interaction between GmHSP17.1 and GmRIP1 was confirmed through Y2H, BiFC and pull-down assay (Figure 6). Recently, our team has reported a role of a sHSP, GmHSP17.9, in nodule development and nitrogen fixation through interacting with GmNOD100, a sucrose synthase specifically induced in the nodules of soybean (Yang et al., 2021). GmHSP17.1 and GmHSP17.9 were both cytosolic proteins, while belonged to different subfamily of sHSPs with GmHSP17.1 in CI subfamily and GmHSP17.9 in CII subfamily (Figure 2D; Yang et al., 2021). Previously, in *Arabidopsis*, the expression of either cytosolic class I or II sHSPs were suppressed using RNAi approach and they showed distinct biochemical behavior *in vitro* and *in vivo* (McLoughlin et al., 2016). These data indicated that individual sHSPs in different subfamilies may regulate nodule development through its own signaling pathways.

Leghemoglobins, an oxygen carrier, required for nitrogenase activity and biological nitrogen fixation in nodules (Jiang et al., 2021). An autooxidation of leghemoglobins and the high rates of respiration were the major source of ROS in nitrogen-fixing nodules of legume plants. Legume nodules were always at the risk of ROS damage, therefore, high capacities of antioxidative proteins were present to apparently protect nodules from oxidative damage (Halliwell and Gutteridge, 1986; Santos et al., 2001; Gunther et al., 2007; Rubio et al., 2009). However, when tightly controlled at low concentrations, ROS also as signaling molecular perform essential roles in rhizobium infection process, nodule development, and nitrogen fixation. Therefore legume nodules are endowed with antioxidant enzymes, such as catalase, ascorbate peroxidase, glutathione peroxidase, and thioredoxins, to control ROS bioactivity (Jamet et al., 2003; Becana et al., 2010; Puppo et al., 2013). PvNod22 was expressed in nodules peaked at 22 dpi, at which time point the nodules had maximum nitrogen-fixing activity and high level of ROS, thus, PvNod22 conferred protection against oxidative stress in nodules in Common Bean (Rodriguez-Lopez et al., 2019). LjGpx1 and LjGpx3, encoding glutathione peroxidases, highly expressed in nodules of the model legume *Lotus japonicus* were, acted as antioxidant enzymes in nodules preventing oxidative processes at different subcellular sites of vascular and infected cells (Matamoros et al., 2015). In our study, GmRIP1, a peroxidase, was found to be expressed specifically in nodules and total peroxidase (POD) activity was also increased in nodules during nodule development in normal growth condition (Figure 7 and Supplementary Figure 3C). In *GmHSP17.1* overexpressed nodules, peroxidase activity was increased greatly resulting in the reduction of ROS concentration, while the loss of function of *GmHSP17.1* caused reduced peroxidase activity and increased ROS content (Figures 7, 8). These results were consistent with the phenotype of nodules overexpressing or suppressing of *GmHSP17.1*. All these data indicated that GmHSP17.1 acted as an antioxidant chaperone in nodule development and nitrogen fixation *via* interacting with GmRIP1 to regulate ROS level and play important signaling roles.

## Data Availability Statement

The original contributions presented in the study are included in the article/Supplementary Material, further inquiries can be directed to the corresponding author/s.

## Author Contributions

CZ and HD designed the research. ZY and HD conducted all experiments, analyzed the data, and wrote the manuscript. CZ corrected the manuscript. JS, XX, YK, WL, and XL provided suggestions during all the process of experiments. All authors contributed to the article and approved the submitted version.

## Conflict of Interest

The authors declare that the research was conducted in the absence of any commercial or financial relationships that could be construed as a potential conflict of interest.

## Publisher’s Note

All claims expressed in this article are solely those of the authors and do not necessarily represent those of their affiliated organizations, or those of the publisher, the editors and the reviewers. Any product that may be evaluated in this article, or claim that may be made by its manufacturer, is not guaranteed or endorsed by the publisher.
