# Supplementary material for: A Nodule-Localized Small Heat Shock Protein GmHSP17.1 Confers Nodule Development and Nitrogen Fixation in Soybean
Source: Front Plant Sci. 2022 Mar 9;13:838718. doi: 10.3389/fpls.2022.838718 (PMC8959767; doi:10.3389/fpls.2022.838718)
Supplement: Supplementary file 1 [file Data_Sheet_1.PDF]

Supplementary Material

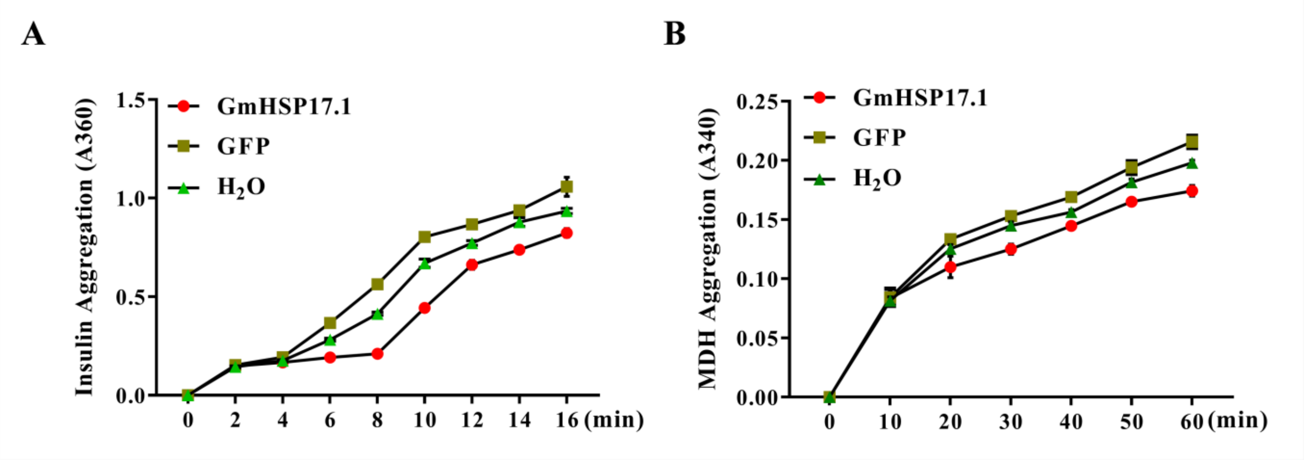

Figure S1. Chaperone activity of GmHSP17.1 *in vitro*. (A) Aggregation of insulin. DTT induced aggregation of insulin was monitored by recording the absorbance at 360 nm. (B) Thermal induced aggregation of MDH. Aggregation of MDH was monitored by recording the absorbance at 340 nm at 45 ℃. H_2O and GFP were used as the negative control. All experiments were repeated at least three times.

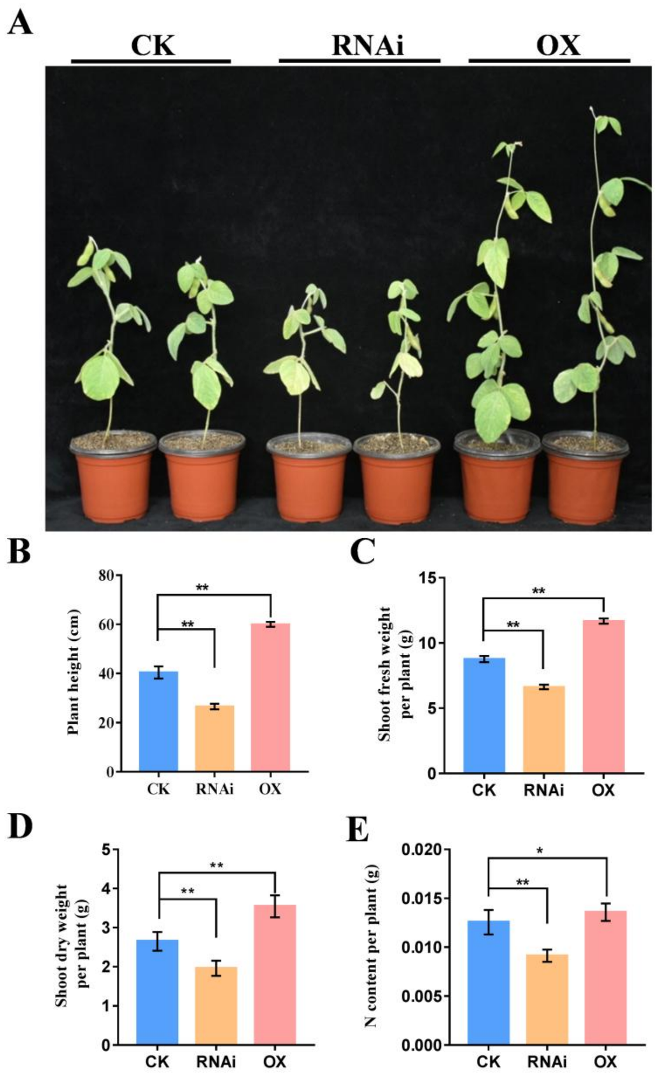

Figure S2. Phenotypic analysis of transgenic composite soybean plants overexpressing and RNAi of *GmHSP17.1*. (A) Growth performance of composite transgenic soybean plants at 28 dpi. (B) Plant height. (C) Shoot fresh weight. (D) Shoot dry weight. (E) N content. CK refers to transgenic plants carrying empty vector. Asterisks indicate statistically significant differences according to Student's *t*-test (two-tailed) (***P*< 0.01), all experiments were repeated at least three times n=10.

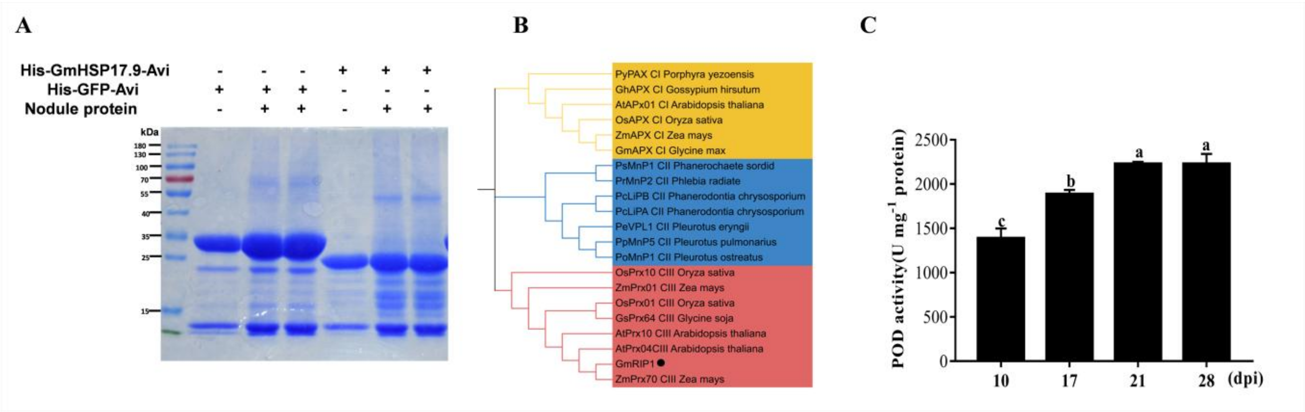

Figure S3. Identification of targets of GmHSP17.1. (A) Isolation of targets of GmHSP17.1 in nodules. The targets were identified by LC-MS/MS after separated on 12% reducing SDS-PAGE. (B) phylogenetic analysis of GmRIP1. Phylogenetic tree was conducted by the MEGA7 software. Accession numbers of the peroxidases are: *Porphyra yezoensis* PyPAX (AAP37478); *Arabidopsis thaliana* AtAPx01 (Q05431); *Gossypium hirsutum* GhAPX (Q05431); *Glycine max* GmAPX(Q05431); *Oryza sativa* OsAPX(P93404); *Zea mays* ZmAPX(Q41772); *Phanerodontia chrysosporium* PcLiPA (AAA53109); *Phanerodontia chrysosporium* PcLiPB (AAA33741); *Pleurotus eryngii* PeVPL1 (AAD01401); *Pleurotus ostreatus* PoMnP1 (AAA84396); *Pleurotus pulmonarius* PpMnP5 (AAX40734); *Phlebia radiate* PrMnP2 (CAC85963); *Phanerochaete sordid* PsMnP1 (BAC06185); *Arabidopsis thaliana* AtPrx04 (Q9LE15); *Arabidopsis thaliana* AtPrx10 (Q9FX85); *Glycine soja* GsPrx64 (KAG4955227); *Oryza sativa* OsPrx01 (Q5VR15); *Oryza sativa* OsPrx10 (Q5U1T3); *Zea mays* ZmPrx01 (A5H8G4); Zea mays ZmPrx70(A5H452). (C) POD activity of nodules at different development stages.

Supplementary Tables

Table S1. List of primers used in this study

Primer name	Sequence (5'to3')	Purpose
GmActin11-F	ATCTTGACTGAGCGTGGTTATTCC	qRT-PCR for Actin11
GmActin11-R	GCTGGTCCTGGCTGTCTCC	
GmHSP17.1-RT-F	GCGGAGAGAGGAGTGTTGAG	qRT-PCR for GmHSP17.1
GmHSP17.1-RT-R	TGCACGGCCTTAACATCAGG	
pHSP17.1-GUS-F	CCCGTCGACGTTACTAGTTGTGTTATTCATG	Promoter-GUS construction
pHSP17.1-GUS-R	CCCGGATCCCTTCCCTTCTTTACTTTACTTGG	
GmHSP17.1-F	CCGGATCCATGAGGCTGGATGGCTTGTG	His-GmHSP17.1-Avi construction
GmHSP17.1-R	CCGAATTCGTTGACCCTTCTGCAATTGC	
GmHSP17.1-YFP^{N}-F	CCCTCTAGAATGTCACTGATACCAAGTTTCTTCG	GmHSP17.1-YFP^{N} construction
GmHSP17.1-YFP^{N}-R	CCCACTAGTACCAGAGATTTGCACGGCCTTAAC	
GmHSP17.1-YFP^{C}-F	CCCTCTAGAATGTCACTGATACCAAGTTTCTTCG	GmHSP17.1-YFP^{C} construction
GmHSP17.1-YFP^{C}-R	CCCACTAGTACCAGAGATTTGCACGGCCTTAAC	
GmRIP1-YFP^{N}-F	CCCGGATCCATGAGGCTGGATGGCTTGTG	GmRIP1-YFP^{N} construction
GmRIP1-YFP^{N}-R	CCCACTAGTGTTGACCCTTCTGCAATTGC	
GmRIP1-YFP^{C}-F	CCCGGATCCATGAGGCTGGATGGCTTGTG	GmRIP1-YFP^{C} construction
GmRIP1-YFP^{C}-R	CCCACTAGTGTTGACCCTTCTGCAATTGC	
GmHSP17.1-BD-F	CCCGAATTCATGTCACTGATACCAAGTTTCTTCG	GmHSP17.1-pGBKT7 construction
GmHSP17.1-BD-R	CCCGGATCCTTAACCAGAGATTTGCACGGCC	
GmHSP17.1-AD-F	CCGAATCCATGTCACTGATACCAAGTTTCTTCG	GmHSP17.1-pGADT7 construction
GmHSP17.1-AD-R	AAGGATCCTTAACCAGAGATTTGCACGGCC	
GmRIP1-BD-F	CCCGAATTCATGAGGCTGGATGGCTTGTG	GmRIP1-pGBKT7 construction
GmRIP1-BD-R	CCCGGATCCGTTGACCCTTCTGCAATTGC	
GmRIP1-AD-F	AAAGAATTCATGAGGCTGGATGGCTTGTG	GmRIP1-pGADT7 construction
GmRIP1-AD-F	AAAGGATCCGTTGACCCTTCTGCAATTGC	
GmHSP17.1-OX-F	CCCGTCGACATGTCACTGATACCAAGTTTC	Overexpression construction
GmHSP17.1-OX-R	AAAGGATCCACCAGAGATTTGCACGGCC	
GmHSP17.1-GFP-F	CCCTCTAGAATGTCACTGATACCAAGTTTC	Subcellular localization for GmHSP17.1
GmHSP17.1-GFP-R	AAAGGATCCTACCAGAGATTTGCACGGCC	
GmHSP17.1-RNA-F	GGGGTACCACTAGTGCGGAGAGAGGAGTGTTGAG	RNAi construction
GmHSP17.1-RNAi-R	GGGGATCCGAGCTCTGCACGGCCTTAACATCAGG	

Table S2. Potential targets of GmHSP17.1 in nodules identified by LC-MS/MS

Potential targets of GmHSP17.1	
**Protein ID**	**Gene ontology (biological process)**
Glyma.14G201700.1.p	response to oxidative stress
Glyma.05G121600.1.p	intracellular sequestering of iron ion
Glyma.13G272000.1.p	calcium ion transmembrane transport
Glyma.13G145100.3.p	ethylene-activated signaling pathway
Glyma.12G032300.2.p	response to oxidative stress
Glyma.10G198800.1.p	nodulation
Glyma.06G216500.1.p	nodulation
Glyma.18G195900.1.p	fatty acid biosynthetic process
Glyma.17G258700.2.p	protein folding
Glyma.17G239700.3.p	biosynthetic process
